# Dosimetric Comparison of Lung-Sparing Radiation Therapy between Volumetric Arc Therapy and Helical Tomotherapy for Unresectable Malignant Pleural Mesothelioma

**DOI:** 10.1155/2019/4568958

**Published:** 2019-12-20

**Authors:** Berrin Pehlivan, Kansu Sengul, Abdullah Yesil, Nilgul Nalbant, Osman Ozturk, Yurday Ozdemir, Erkan Topkan

**Affiliations:** ^1^Department of Radiation Oncology, Bahcesehir University, Goztepe, Istanbul, Turkey; ^2^Medstar Antalya Hospital, Department of Radiation Oncology, Antalya, Turkey; ^3^Baskent University Medical Faculty, Department of Radiation Oncology, Adana, Turkey

## Abstract

**Objective:**

To compare volumetric arc therapy (VMAT) and helical tomotherapy (HT) plans in terms of dosimetric parameters in positron emission tomography- (PET-) computerized tomography- (CT-) based radiation therapy planning in unresectable malignant pleural mesothelioma (MPM).

**Methods:**

CT and coregistered PET-CT data from seven patients with histologically-proven MPM were utilized for VMAT and HT plans. Target volumes and organs at risk (OARs) were delineated. The prescription doses for planning target volume 1 (PTV_1_) and PTV_2_ were 45.0 Gy and 54 Gy in 1.8 Gy/fr, respectively. Each technique was evaluated in terms of target volume coverage and OAR doses.

**Findings:**

Although the maximum (*p*=0.001) and mean (*p* < 0.001) doses of PTV_1_, and PTV_2_ (*p* < 0.001 for maximum and *p*=0.001 for mean doses) favored the HT technique over VMAT, both techniques efficiently covered the target volumes. Additionally, HT also provided more homogeneous dose distribution (*p* < 0.001) and numerically lower doses received by most OARs, but again both rotational techniques were successful in keeping the OAR doses below the universally accepted limits. The major disadvantage of the HT technique was the requirement for longer treatment times (7.4 versus 2.5 minutes/fr; *p* < 0.001) to accomplish the intended treatment.

**Conclusion:**

Results of this dosimetric comparison clearly demonstrated the possibility of safe hemithoracic irradiation of medically/technically unresectable MPM patients with either of the two rotational RT techniques, namely the VMAT and HT. Clinically, considering their poor prognosis, these promising findings may open a potential new window for curative treatment of unresectable MPM patients, if further confirmed by future clinical studies.

## 1. Background

Malignant pleural mesothelioma (MPM) is a rare, but commonly fatal tumor. Despite the fact that extrapleural pneumonectomy (EPP) was viewed as the surgical standard for MPM, results of the Mesothelioma and Radical Surgery (MARS) trial challenged this tradition in favor of the pleurectomy/decortication (P/D) procedure [1, 2]. Taioli and colleagues' recent meta-analysis additionally exhibited that the less invasive P/D provides improved survival times and quality of life measures than the EPP [3]. Along these lines, with its favorable toxicity profile, the P/D and chemotherapy turned into the current widely preferred treatment of choice for the MPM patients. But unfortunately, overall 85–90% of patients present with technically or medically unresectable tumors due to various reasons, such as poor execution status, comorbid illnesses, and/or extensive tumor burden [4, 5].

Until recently, barring the hemithoracic irradiation after EPP, the primary role of radiotherapy (RT) was limited to prophylactic irradiation of the drain sites and palliation of symptomatic lesions in MPM. RT planning (RTP) for post-EPP hemithoracic RT is relatively straightforward with two traditional opposing photon beam fields and electron boost fields if required, which permits better sparing of the contralateral intact lung. However, because of the high risk of severe or even fatal acute and late pulmonary toxicities, this technique is almost never applicable for unresectable MPM for patients with bilateral intact lungs. Induction chemotherapy may alternatively increase the resection rates on a hypothetical basis, yet considering the fact that the response to induction chemotherapy is usually far less than the prerequisites for any type of curative surgery, RT still stays to be the main potential for cure or palliation in most MPM patients [6]. RTP with the conventional photon and electron combinations and even with the more sophisticated intensity-modulated RT (IMRT) and image-guided RT (IGRT) techniques may be difficult in inoperable patients or those treated with upfront P/D due to the large and irregularly shaped tumoral involvement in the thorax. In this manner, these patients require high RT doses to improve local control rates that oppose limited lung tolerance to RT, increased low-dose bath volumes in the contralateral lung caused by dose spreading, and the close proximity of radiosensitive healthy structures such as the liver, ipsilateral kidney, heart, spinal cord, and esophagus. Volumetric arc therapy (VMAT) and helical tomotherapy (HT) appear to be promising in this therapeutic challenge by their selective dose distribution between the target volumes and organs at risk (OARs) with almost no compromise on the costodiaphragmatic recess and/or pericardium doses [7–10].

To our best information, negating the patients' requirement for alternative RT options and great innovations in diagnostic and RTP methodologies, to date no studies have compared the promising VMAT and HT dosimetrically in the setting of post-P/D or unresectable MPM where both lungs remain in place. Therefore, we aimed to compare the VMAT and HT techniques dosimetrically with regards to the tumor coverage and OAR doses.

## 2. Materials and Methods

We reviewed our patients' records to identify stage T_2-4_N_0-3_M_0_ MPM patients as per the AJCC-7 staging system [11] who were not candidates for a curative resection due to medical or technical reasons. This pure dosimetric comparison study was approved by the institutional ethics committee before collection of any patient information.

Seven patients with unresectable MPM who underwent positron emission tomography-computerized tomography (PET-CT) imaging in the supine position with both arms raised over their heads utilizing the T-bar to mimic set-up conditions were selected. The PET-CT scans were performed in an integrated PET-CT system (Discovery-STE 8, General Electric Medical Systems, Milwaukee, WI, USA) using a previously published imaging procedure [12]. PET-CT data sets were transferred to a treatment planning system (Eclipse 10.0, Varian Medical Systems, Palo Alto, CA, USA) and HT HI-ART2 in the DICOM format. Fused images were utilized for RTP. VMAT plans were developed using Eclipse V10.0 with double arc, clockwise/counter-clockwise with 30 and 330° collimators. For all HT plans the “inverse planning” system was utilized. During the planning process, first, the parameters, including the field width, pitch, modulation factor (MF), importance, and penalties for all structures were determined. The radiotherapy physicist in charge was free to choose one of the three field widths of 1.0, 2.5, and 5.0 cm as appropriately, depending on the target size. The chosen value specified the size of the field in the machine isocenter along the longer axis of the patient. Similarly, the pitch parameter specified the table motion during one full gantry rotation according to the part of the field width. In order to obtain a homogeneous dose distribution at anywhere outside the isocenter, this value was set to mandatorily meet the condition of 0.86/*n*, where *n* was an integer.

Target volumes were defined both on the diagnostic CT and integrated PET-CT images by one of the two radiation oncologists with specific experience in RTP of MPM. The window and level for the PET images were set according to a method previously described by Erdi et al. [13]. In this protocol, the hottest pixel value in the lesion was measured and the upper- and lower-window levels were set to this measured maximum and to 42% of the maximum, respectively. Gross tumor volume (GTV) was defined as the volume encompassing the measurable primary tumor and the involved hilar and/or mediastinal lymph nodes recognized on either the PET-CT or diagnostic CT. The entire ipsilateral hemithorax was defined as the clinical target volume (CTV) which encompassed the hemithoracic visceral and parietal pleura along the ribs and pericardium from the superior thoracic inlet to the lowest level of diaphragm insertion at the level of the L1-2 vertebral body and GTV plus 1-cm margin at all directions respecting the natural anatomical barriers and uninvolved OARs, such as the vertebral column and heart. Planning target volume (PTV1) encompassed the CTV + 1-cm margin at all directions, while the boost PTV (PTV2) was defined as the original CTV as defined above. Clinical target volume (CTV) was created automatically with a 1-cm margin around the GTV in all dimensions with respect to the natural anatomical barriers and uninvolved OARs, such as the vertebral column and heart.

Diagnostic CT data sets were utilized for delineation of OARs due to the difficulties in defining the anatomic borders of organs with PET. For each patient, the OARs included the right and left lungs, total lung, liver, heart, esophagus, spinal cord, and right and left kidneys. For planning purposes, the Quantitative Analyses of Normal Tissue Effects in Clinic (QUANTEC) radiation dose constraints were applied [14]. Accordingly, 50 Gy maximum point dose for the spinal cord, 18 Gy mean dose for the kidneys, V_30_ (volume receiving 30 Gy) < 50% and mean dose < 28–32 Gy for the liver, V_35_ < 50%, V_50_ < 40%, and mean dose < 34 Gy for the esophagus, and mean dose < 26 Gy for the heart were allowed. The intended lung dose limitations were as follows: total mean lung dose ≤ 21 Gy, V_20_ ≤ 40%, and contralateral lung V_20_ ≤ 7%.

The prescription doses for PTV_1_ and PTV_2_ were 45.0 Gy (1.8 Gy/fr, 25 daily fractions) and 54 Gy (1.8 Gy/fr, 30 daily fractions), respectively. The primary end point was to deliver the prescribed doses to ≥95% of the each PTV without sacrificing OAR dose constraints. For this purpose, the created dose volume histograms were utilized for comparisons between the VMAT and HT plans in terms of the PTV coverage and OAR doses. Comparisons between the VMAT and HT plans were performed by analyzing mean, V_5_ (volume receiving 5 Gy), V_10_, and V_20_ doses for each lung and mean and V_20_ doses for the total lung, mean dose for the heart, mean doses for each kidney, maximum dose for the spinal cord, and mean and V_30_ doses for the liver. Conformity index (CI), homogeneity index (HI), total monitor units, and total treatment times were calculated for further comparisons. The CI and HI were defined as follows:

(1) $$\begin{array}{c} \text{CI}=\frac{\text{VRI}}{\text{TV}}, \end{array}$$

VRI: volume of prescribed dose for PTV; TV: total volume of PTV.

(2) $$\begin{array}{c} \text{HI}=\frac{I_{\max}}{\text{RI}}, \end{array}$$

*I*_max_: maximum dose; RI: prescribed dose for PTV.

## 3. Results

**Figure 1:**
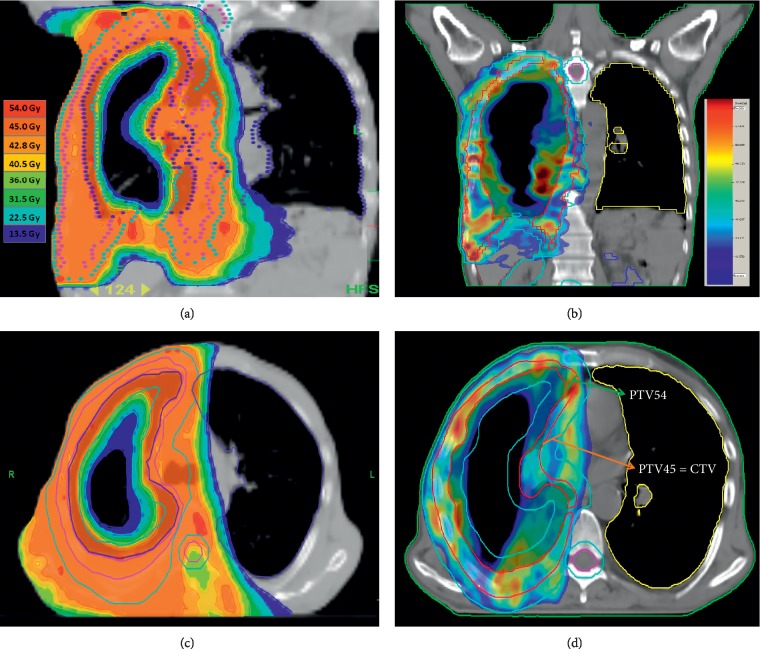
Dose distributions in coronal views. (a) Helical tomotherapy (HT), (b) volumetric arc therapy (VMAT), and dose distributions in axial views (c) HT and (d) VMAT. PTVX: planning target volume receiving X dose and CTV: clinical target volume.

Patient characteristics are shown in Table 1. Respective dosimetric outcomes of the PTVs and OARs for each technique are as shown in Table 2 and Figure 1, respectively. For all plans, as intended 95% of PTVs were covered with at least 95% of the prescribed dose with no significant differences between the two techniques with regards to the minimum doses of PTV_45_ (*p*=0.81) and PTV_54_ (*p*=0.65). However, the respective PTV_45_ maximum (53.9 versus 48.4 Gy; *p*=0.001) and mean (49.2 versus 45.2 Gy; *p* < 0001) and PTV_54_ maximum (64.3 versus 57.8 Gy; *p* < 0.001) and mean (58.6 versus 55.0 Gy; *p*=0001) doses favored the HT plan over the VMAT. Likewise, albeit the CI of both plans were similar (0.96 versus 098; *p*=0.74), the HT plan provided significantly more homogeneous dose distribution than did the VMAT plan (HI: 1.1 versus 1.036; *p* < 0.001).

Considering the OAR doses, the ipsilateral lung mean (33.5 versus 38.2 Gy; *p*=0.007) and median V_10_ (95.1% versus 100%; *p*=0.028) doses were significantly lower with HT than with the VMAT plan. The median contralateral lung V_5_ (30.6% versus 67.8%; *p*=0.001), V_10_ (29.6% versus 51.4%; *p*=0.009), and V_20_ (0.5% versus 3.5%; *p*=0.02) doses were likewise significantly lower in HT plans. Similarly, the total lung mean (16.1 versus 19.8 Gy; *p*=0.003) and V_20_ (29.3 versus 36.2%; *p*=0.021) doses also favored the HT over the VMAT plans in a significant manner. However, HT plans appeared to be preferable, yet both plans were judged to be acceptable for clinical use as the lung constraints were met with the either technique with regards to the QUANTEC recommendations for curative lung irradiation.

The other OAR doses, namely the maximum spinal cord (*p*=0.005); heart mean (*p*=0.006), V_25_ (*p*=0.002), and V_30_ (*p*=0.001) doses; and esophageal mean (*p*=0.001), V_35_ (*p*=0.017), and V_50_ (*p*=0.024) doses were also significantly better with HT than with VMAT plans, as depicted in Table 2. On the other hand, the mean uni- and contralateral kidney and liver doses were comparably similar between the two techniques. Again both plans were judged to be clinically applicable as the OAR constraints were successfully met by both techniques for each organ according to the QUANTEC's OAR dose specifications. Further comparisons between the two techniques uncovered that the relative advantages provided by the HT technique came with the cost of higher median monitor units (6590 versus 529 monitor units/fr; *p* < 0.001) and, therefore, longer treatment times (7.4 versus 2.5 minutes/fr) compared with the VMAT procedure.

## 4. Discussion

The outcomes of the present dosimetric comparison in unresectable MPM patients (*n* = 7) with two intact lungs exhibited that, in spite of the fact that the ipsi-, contralateral, and total lung and most OAR radiation doses were altogether notably lower in HT than the VMAT designs, both rotational RT techniques were successful in creating RTPs applicable to clinical practice in terms of target volume coverage and OAR doses defined by the QUANTEC report. However, our encouraging results also revealed that these relative advantages of HT plans came at the cost of significantly longer irradiation times.

Past investigations clearly demonstrated that both the VMAT and HT procedures were highly efficient to create excellent dose distributions in numerous tumor types, including the MPM [7–10, 15, 16]. In the most recent decade, paralleling with the wide acknowledgment of P/D as a similarly compelling but more conservative surgical approach than the EPP, the use of RT for positive/potentially positive tumor sites has increased to a large extent as an adjuvant to P/D. In one such study, Rimner et al. investigated the feasibility of hemithoracic intensity-modulated pleural RT (IMPRINT) in the setting of postchemotherapy and P/D; of the eligible 26 patients, grades 2-3 radiation-induced pulmonary toxicity was reported in only 30.8% cases with no grades 4-5 toxicity instances [16]. The median progression-free and overall survival (OS) durations were 12.4 and 23.7 months, respectively with encouraging respective 2-year OS rates of 59% and 25% in patients with resectable and unresectable tumors [16]. Rimner et al. further compared the post-P/D HT with 3-dimensional conformal RT (3D-CRT) plans and demonstrated that the target coverage was notably improved with HT plans, which further translated into significantly improved local recurrence-free survival durations (19.0 versus 10.9 months; *p* < 0.05) [17]. Kishan et al. investigated the feasibility of intensity-modulated proton therapy (IMPT) in MPM patients with both lung intact and noted that IMPT was efficient in reducing the mean RT doses to the uninvolved lung, heart, esophagus, liver, and ipsilateral kidney [18]. Moreover, the researchers also reported superior sparing of the uninvolved lung even when further mediastinal boost doses were required for node-positive disease status. Although the results of our present study appear to confirm these studies regarding the efficiency of sophisticated RT techniques in MPM, it differs from them in at least in two ways: first, no previous dosimetric study directly compared the two rotational techniques with each other. And second, the metabolic PET-CT based RTP was not utilized in neither of the accessible investigations despite of its widely recognized very high sensitivity and specificity rates in MPM [19, 20].

Despite of the proven efficacy of IMRT in MPM patients with intact lungs, the disappointingly high fatal pulmonary toxicity rates, as high as 46%, hampers its routine use in radiation oncology clinics [21–24]. These unacceptable high fatal complication rates after hemithoracic RT with no doubt underline the commitment for the sparing of the ipsi- and contralateral healthy lung tissues at least down to the RT constraints recommended for lung cancer irradiation, in order to achieve better clinical outcomes in a more secure way. In this regard, although the HT procedure provided comparatively better plans than the VMAT procedure, our present dosimetric results accomplished with either the HT or VMAT technique are encouraging with regards to the efficient sparing of the normal lung tissues, which is the principal end goal of any RT technique for MPM irradiation. With specific emphasis on the dose constraints recommended by the QUANTEC for partial lung irradiation, both the mean (16.1 Gy for HT and 19.8 Gy for VMAT) and V_20_ (29.3% for HT and 36.2% Gy for VMAT) of total lung, and contralateral lung V20 (0.5% for HT and 3.5% Gy for VMAT) doses observed here in both techniques meet the predefined risk of less than 20% symptomatic pneumonitis, separately. Similarly, these results are also accordant with the respective values of mean < 20 Gy and V_20_ ≤ 37% for total lung dose constraints specified in the landmark lung cancer dose escalation study of the RTOG 0617 protocol [25]. Moreover, loaning support on the past investigations, the results of our dosimetric comparison likewise displayed that both the HT and VMAT procedures were also capable of keeping the OAR doses below the prespecified values by QUANTEC proposals, other than the lung doses.

Escalated RT doses may potentially improve local control rates which may translate into a survival advantage in nonmetastatic MPM patients [26]. Underscoring the importance of the total RT dose, even for palliative purposes, Ball et al. demonstrated that effective symptom relief was achievable only in 4% of patients with <40 Gy, which increased to 66% with doses >40 Gy [27]. Albeit the evidence of potentially improved tumor control rates come from 3D-CRT studies, in general it is not conceivable to escalate RT doses beyond the conventional limits due to the severe toxicity concerns. In this regard, Maggio et al. in their HT study investigated whether a safe escalated dose to the pleural space and PET/CT-positive areas was achievable in patients with unresectable MPM [28]. Their outcomes clearly proved that the HT procedure was able to safely escalate the total dose to at least 62.5 Gy in PET-positive regions, while treating the pleural cavity with 56 Gy in 25 fractions with no significant trade-off in OAR doses. Although the possibility of dose escalation was beyond the scope of our present study, confirming Maggio's outcomes, our results also suggested the possibility for dose escalation in such patients with use of rotational IMRT techniques.

Despite of HT procedure's notable dosimetric superiority over VMAT in some measures, the HT technique also has two major drawbacks. First, because of the need for longer irradiation times, HT is without any doubt more vulnerable to inherent respiratory movement problems mandating more careful setup and online imaging procedures. However, we believe that this is only a matter of time which will probably be solved in the near future with the improvements in HT technology. And second, as the HT technique requires relatively longer treatment times for each RT fraction compared with VMAT (7.4 versus 2.5 minutes; *p* < 0.001), its feasibility may be questioned by some authors for the case of MPM. Rationally, although the 7.4 minutes is a tolerable time for most patients in the sense of a general radiation oncology practice, it may yet be problematic for some patients with limited tolerance capacity. Furthermore, longer treatment times may be disadvantageous in heavily loaded radiation oncology clinics. Nonetheless, it is again rational to foresee or at least hope that this problem will also be soon negated with the rapid innovations in RT technologies.

## 5. Conclusions

Although HT appeared to provide better dosimetric measures than the VMAT procedure in some specific end goals, the results of this comparative dosimetric study clearly demonstrated the possibility of safe hemithoracic irradiation of medically or technically unresectable MPM patients with either of the two rotational RT techniques. Clinically, considering their poor prognosis, these findings may potentially be promising for curative treatment of MPM patients. However, the clinical translations of these promising dosimetric outcomes ought to be confirmed by further clinical studies before their recommendation for routine clinical use.

## Figures and Tables

**Table 1:** Patient characteristics.

Patients	Gender	Age	Laterality	Stage
1	Male	72	Left	T_1a_N_2_M_0_
2	Male	51	Right	T_4_N_3_M_0_
3	Female	54	Right	T_2_N_0_M_0_
4	Male	43	Right	T_4_N_1_M_0_
5	Female	40	Left	T_3_N_0_M_0_
6	Male	68	Right	T_3_N_0_M_0_
7	Female	43	Left	T_4_N_1_M_0_

**Table 2:** Dose volume parameters for planning target volumes and organs at risk.

Characteristics	VMAT median (range)	HT median (range)	*p* value
PTV_45_, Gy			
*D*_max_	53.9 (52.2–59.2)	48.4 (47.1–49.8)	0.001
*D*_min_	38.8 (30.7–43.3)	37.5 (19.4–43.7)	0.81
*D*_mean_	49.2 (48.1–52.1)	45.8 (45.6–46.1)	<0.001

PTV_54_, Gy			
*D*_max_	64.3 (61.9–69.9)	57.8 (56.2–59.2)	<0.001
*D*_min_	46.8 (37.2–52.1)	45.4 (26.7–51.7)	0.65
*D*_mean_	58.6 (56.3–61.9)	55.0 (54.7–55.2)	0.001

Ipsilateral lung			
Mean dose, Gy	38.2 (20.8–51.4)	33.5 (18.0–40.1)	0.007
V_5_ (%)	100 (96.2–100)	99.7 (98.0–100)	0.59
V_10_ (%)	100 (77.2–100)	95.1 (68.2–100)	0.028
V_20_ (%)	92.0 (43.6–100)	80.2 (41.2–100)	0.10

Contralateral lung			
Mean dose, Gy	11.8 (5.2–18.1)	6.7 (5.3–8.1)	0.018
V_5_ (%)	67.8 (33.2–90.0)	30.6 (21.4–49.0)	0.001
V_10_ (%)	51.4 (3.9–100)	29.6 (11.9–49.0)	0.009
V_20_ (%)	3.5 (0–55.0)	0.5 (0–14.3)	0.002

Total lung			
Mean dose, Gy	19.8 (17.8–33.0)	16.1 (10.5–24.4)	0.003
V_20_ (%)	36.2 (25–69.0)	29.3 (13.08–45.5)	0.021

Spinal cord, Gy			
*D*_max_	39.3 (31.9–44.2)	33.2 (22.5–34.2)	0.005

Heart			
Mean dose, Gy	33.3 (12.1–54.9)	25.8 (7.3–45.7)	0.006
V_25_ (%)	57.9.0 (7.21–100)	43.5 (4.1–95.3)	0.002
V_30_ (%)	50.9 (4.45–100)	34.8 (1.4–88.9)	0.001

Mean kidney dose, Gy			
Ipsilateral	2.5 (1.3–11.2)	1.6 (0.7–6.4)	0.28
Contralateral	1.9 (0.3–8.9)	2.0 (0.2–5.2)	0.27

Liver			
Mean dose, Gy	13.1 (6.3–33.6)	15.1 (8.3–31.4)	0.31
V_30_ (%)	11.0 (0.0–59.2)	13.3 (0–50.8)	0.67

Esophagus			
D_mean_	38.6 (16.2–57.8)	29.9 (8.3–44.2)	0.001
V_35_	57.2 (0.9–100)	42.3 (0–100)	0.017
V_50_	40.3 (0–98.9)	29.6 (0–97)	0.024

Dosimetric indices			
Conformity index	0.96 (0.93–0.99)	0.98 (0.97–0.99)	0.74
Homogeneity index	1.1 (1.1–1.1)	1.036 (1.03–1.05)	<0.001

Treatment duration			
Monitor units, per fr	529 (471–765)	6590 (6185–7116)	<0.001
Treatment time (min/fr)	2.5 (2.1–3.0)	7.4 (6.9–7.8)	<0.001

PTV: planning target volume; V_X_: volume receiving X Gy; *D*_max_: maximum dose; *D*_min_: minimum dose; *D*_mean_: mean dose; fr: fraction.

## Data Availability

The datasets used and analyzed during the current study are available from the corresponding author on reasonable request.
